# Progress of Research on the Regulatory Pathway of the Plant Shade-Avoidance Syndrome

**DOI:** 10.3389/fpls.2020.00439

**Published:** 2020-04-15

**Authors:** Xiaoyan Wang, Xinqiang Gao, Yuling Liu, Shuli Fan, Qifeng Ma

**Affiliations:** ^1^College of Biology and Food Engineering, Anyang Institute of Technology, Anyang, China; ^2^State Key Laboratory of Cotton Biology, Institute of Cotton Research of CAAS, Anyang, China; ^3^Research Base, Anyang Institute of Technology, State Key Laboratory of Cotton Biology, Anyang, China

**Keywords:** shade-avoidance syndrome, photoreceptors, phytochrome-interacting factors, phytohormones, signaling mechanisms

## Abstract

When subject to vegetational shading, shade-avoiding plants detect neighbors by perceiving reduced light quantity and altered light quality. The former includes decreases in the ratio of red to far-red wavelengths (low R:FR) and low blue light ratio (LBL) predominantly detected by phytochromes and cryptochromes, respectively. By integrating multiple signals, plants generate a suite of responses, such as elongation of a variety of organs, accelerated flowering, and reduced branching, which are collectively termed the shade-avoidance syndrome (SAS). To trigger the SAS, interactions between photoreceptors and phytochrome-interacting factors are the general switch for activation of downstream signaling pathways. A number of transcription factor families and phytohormones, especially auxin, gibberellins, ethylene, and brassinosteroids, are involved in the SAS processes. In this review, shade signals, the major photoreceptors involved, and the phenotypic characteristics of the shade-intolerant plant *Arabidopsis thaliana* are described in detail. In addition, integration of the signaling mechanisms that link photoreceptors with multiple hormone signaling pathways is presented and future research directions are discussed.

## Introduction

Sunlight is the energy source for plant growth. The spectrum of solar radiation utilized by green plants for conducting photosynthesis is termed photosynthetically active radiation (PAR; 400–700 nm). When PAR or light quality is lower than a certain saturation (Morgan and Smith, 1978), plants receive optical signals caused by canopy shade. To reduce the degree to which they are affected by the shade, a series of responses termed the shade-avoidance syndrome (SAS) is triggered (Morgan and Smith, 1978; Smith, 1982; Smith and Whitelam, 1997).

**Figure 1:**
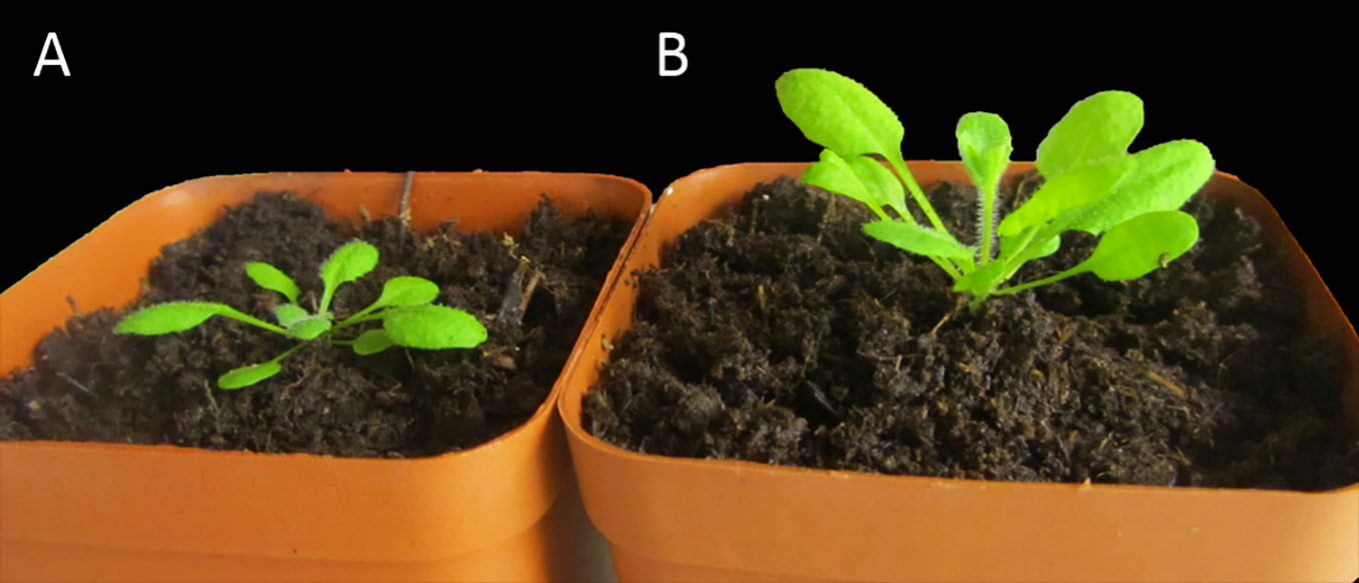
Phenotype of *Arabidopsis* plants grown under low or high red: far-red light (R:FR) ratio. **(A)** Phenotype of *Arabidopsis* plant grown in an open environment under white light. **(B)** Phenotype of *Arabidopsis* plant grown under high-density canopy shade (R:FR ratio 0.2–0.4).

During the evolution of plants, selective advantages have led to phenotypic differences among species. Some shade-tolerant plants, such as *Alocasia*, have thin leaves that contain a high chlorophyll content. Their leaf epidermal cells, similar to a camera lens, focus light on the mesophyll tissues so that weak light can be utilized to conduct effective photosynthesis (Middleton, 2001). However, with respect to the shade-intolerant *Arabidopsis thaliana*, at the seedling stage, hypocotyls and stems growing in a shaded environment are severely elongated. The cotyledon and early true leaves grow at a higher position on the stem to weaken the degree to which the plants are shaded. At the rosette stage, the shade signals result in upward bending of the cotyledon and true leaves, which is termed hyponasty. As a result of hyponasty, the leaf lamina is placed at a higher, more favorably lit position (**Figure 1**). In addition, shade signals weaken expansion of the leaf lamina but strengthen petiole elongation. Longer petioles enhance the amplitude of fluctuation in blade position to avoid the shade environment caused by surrounding plants (Casal, 2012). During the period of cauline leaf growth, shade signals result in earlier flowering and fewer branches. As for vegetative *Arabidopsis*, it typically grows similar to a rosette, and elongation of the internode and generation of cauline leaves is associated with subsequent reproductive growth and development. Accelerated flowering allows plants to complete the life cycle quickly to reduce the chance of future shade. Reduction in branching is an additional response of plants to avoid shade, because in *Arabidopsis* prolific development of branches from the basal rosette will undoubtedly increase the proportion of shaded tissues.

In this manner, immovable plants can adjust their growth strategy and change their spatial configuration to capture greater amounts of sunlight and occupy a larger spatial area during competition with surrounding plants (Ballaré et al., 1990). Undoubtedly, under high-density planting, some responses of shade-avoidance, such as reorientation of leaves toward more light, are beneficial to plants. However, some effects on the perception of shade signals, such as elongation growth and accelerated flowering at inappropriate phases, may be detrimental for yield (Donohue et al., 2001; Kebrom and Brutnell, 2007). Although breeding programs have led to improved performance of new cultivars under high planting density, many crops remain sensitive to and responsive to canopy shade. Here, based on recent progress in understanding the SAS, shade signals and key regulatory factors are reviewed, mostly focusing on *Arabidopsis*. In addition, current knowledge of the signaling mechanisms linking several photoreceptors with a variety of hormone signaling pathways is discussed.

## Perception of Shade Signals by Photoreceptors

### Light Signaling

Light signaling refers to the alterations of surrounding light conditions perceived by the plant photoreceptors. When the vegetation is dense, the majority of red light (R; λ = 600–700 nm) and blue light (B; λ = 400–500 nm) is preferentially absorbed by crop leaves at a higher position. The reflected or transmitted light is enriched in the green (G; λ = 500–580 nm) and far-red (FR; λ = 700–800 nm) spectral regions, leading to a decrease in the ratio of R:FR (low R:FR) and low blue light (LBL). Plants perceive these shade signals through multiple photoreceptors, which in turn initiate signaling cascades to cause the SAS (Morgan and Smith, 1978; Smith, 1982; Smith and Whitelam, 1997).

Under normal conditions, R:FR is approximately 1.2–1.5 at midday, varying little with season or weather conditions. Underneath the vegetation canopy, the value can be as low as 0.05 (Smith, 1982). On the basis of previous research, four approaches can be adopted to simulate and study the shade signal. As early as 1978, by ensuring PAR, Morgan and Smith (1978) added far red light to white light to reduce R:FR and applied treatments to study the SAS in plants. The PAR can be provided artificially or through sunlight, and in this manner, the plant can be exposed to light of the ideal R:FR ratio. The second approach is realized by applying a pulse of far-red light at the end of the daily photoperiod. To achieve the expected results, this brief decrease in R:FR must be drastic. Given that R:FR fluctuates during the day, slight variation in R:FR may be ineffective. In addition, the light of certain wavebands can be reduced with color filters placed above the plant or around the stem (Yanovsky et al., 1995). Finally, using a genetic approach, mutants with optical-signal defects can be treated to observe the physiological and molecular outputs under real sunlight and shade light conditions (Sellaro et al., 2010; Sellaro et al., 2011). Although some of the afore-mentioned experiments may encounter technical difficulties in accurately simulating the natural environment, such experimental conditions are conducive to study the SAS.

### Photoreceptors

At least five classes of photoreceptors in plants are recognized. These classes comprise phytochromes that absorb red and far-red light, cryptochromes that absorb UV-A light (λ = 315–400 nm) in the blue light and near ultraviolet areas, phototropin that absorbs blue light, the ZEITLUPE (ZTL) group of proteins that absorb blue-green light, and UV-B RESISTANCE 8 (UVR8) discovered in 2011 that absorbs UV-B light (λ = 280–315 nm) (Chory, 2010; Rizzini et al., 2011; Losi and Gärtner, 2012).

The two photo-convertible isoforms of phytochromes are the red light-absorbing form (Pr) and far-red light-absorbing form (Pfr). Five phytochrome genes (*PHYA– PHYE*) have been identified in *Arabidopsis* (Franklin and Quail, 2010). *PHYB* plays a prominent role in regulation of the SAS. *Arabidopsis phyB* mutants display a constitutive SAS under normal and high R:FR environments, suggesting that PHYB plays a negative role in the control of the SAS (Reed et al., 1993). PHYB, PHYD, and PHYE function redundantly to regulate leaf morphology and flowering time in response to low R:FR (Aukerman et al., 1997; Devlin et al., 1998; Franklin et al., 2003). Given gene replication, *PHYC* is probably descended from the *PHYA* lineage (Mathews and Sharrock, 1997). PHYA is rapidly degraded in its Pfr form, whereas PHYB–PHYE are all relatively stable in the respective Pfr forms (Bae and Choi, 2008; Franklin and Quail, 2010; Casal, 2013). Previous studies have shown that the SAS induced by PHYB deactivation is gradually antagonized by PHYA, which is intensely induced by low R:FR to inhibit the excessive elongation response of seedlings (Martinez-Garcia et al., 2014). In addition, as the receptor of far-red light, PHYA plays a key role in de-etiolation in FR-rich environments, such as extremely low R:FR (Shinomura et al., 2000; Rausenberger et al., 2011; Casal et al., 2014).

Monocotyledon species harbor three phytochromes, namely PHYA, PHYB, and PHYC (Kay et al., 1989). Maize has two *PHYB* alleles, *PHYB1* and *PHYB2*, which are completely or partially functionally redundant on apical dominance, elongation reaction, and flowering time (Sheehan et al., 2004; Sheehan et al., 2007). Sorghum *phyB1* mutants exhibit SAS phenotypes, such as insensitivity to photoperiod, elongation reaction, low chlorophyll content, and no presentation of de-etiolation under high-intensity red-light radiation (Finlayson et al., 2007; Kebrom et al., 2010). In addition, the rice *phyB* and *phyC* mutants and the double mutants *phyAphyB* and *phyAphyC* all show early flowering and the SAS (Jumtee et al., 2009; Sun et al., 2015).

In *Arabidopsis*, the cryptochrome group includes three genes, namely *CRY1*, *CRY2*, and *CRY3*. Under any light condition, *CRY1* can be detected in the nucleus and cytoplasm, whereas *CRY2* is mainly enriched in the nucleus and is degraded under blue light (Yu et al., 2007). *CRY1* and *CRY2* not only act upstream of *PHYTOCHROME INTERACTING FACTOR 4* (*PIF4*) and *PIF5*, but also physically interact with phytochrome-interacting factors (PIFs) to modulate activities of PIFs to promote growth under low-intensity blue light (Pedmale et al., 2016). Activation of the UVR8 photoreceptor enhances rapid PIF5 degradation *via* the ubiquitin-proteasome system to attenuate plant responses to canopy shade (Hayes et al., 2014; Mazza and Ballaré, 2015; Sharma et al., 2019). In *Arabidopsis*, *ztl* mutants are hypersensitive to red light and ZTL interacts with PHYB and *CRY1* (Somers et al., 2000; Jarillo et al., 2001; Kevei et al., 2006). ZTL is shown to modulate PHYB‐mediated shade signaling *via* an auxin-dependent manner in the wild tobacco *Nicotiana attenuate* (Zou et al., 2019).

## Signaling Pathways of SAS

**Figure 2:**
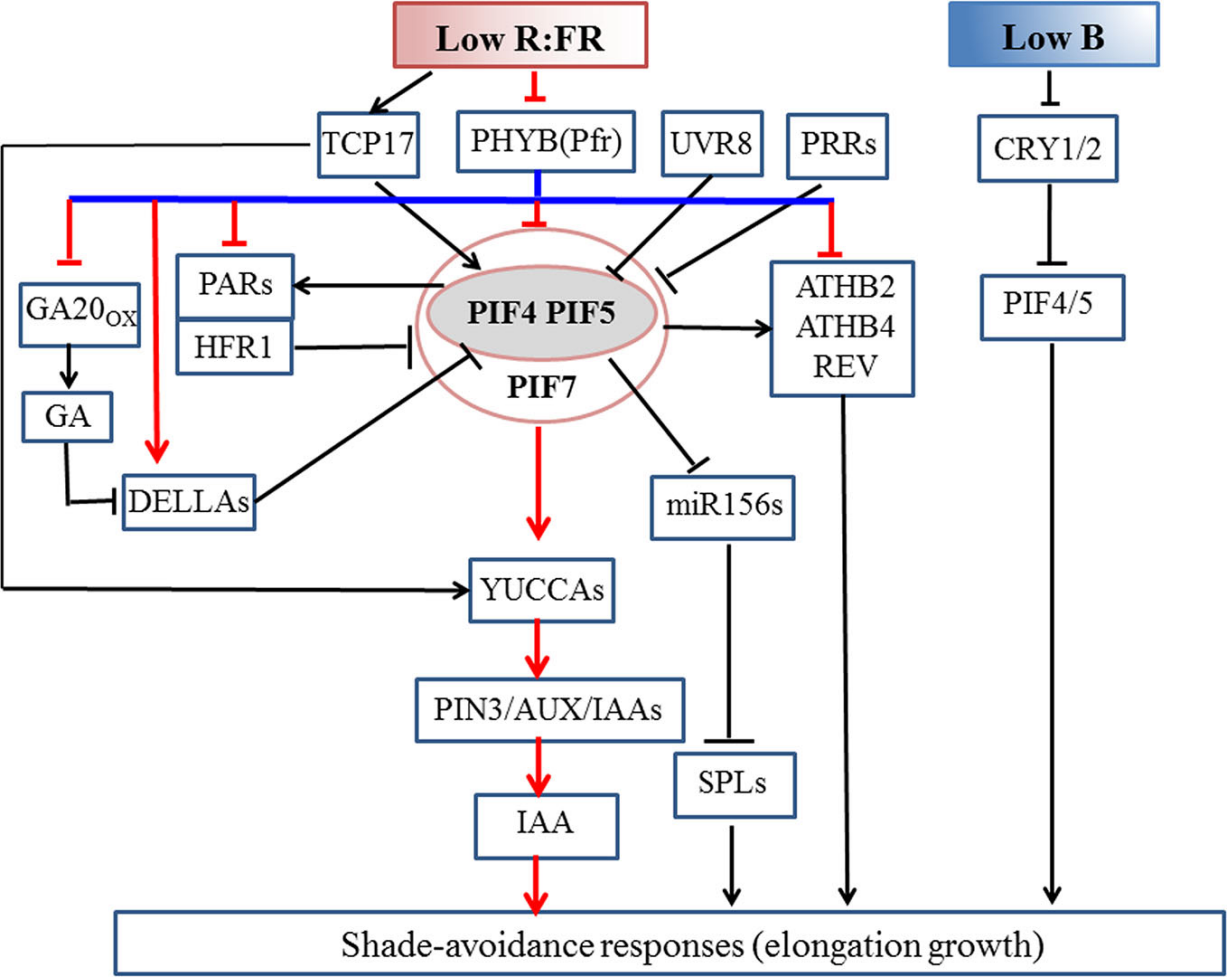
Molecular mechanism of the shade-induced elongation growth in *Arabidopsis thaliana*. Low R:FR enhances the functions of PIF4/5/7 by inhibiting the activity of phyB under shaded conditions. During the early shade response, low R:FR signal activates PIF4/5/7, thus promoting auxin biosynthesis in the cotyledon, which is then transported out to the hypocotyl where it induces cell elongation. Under prolonged shade conditions, PIF4/5/7 modulate IAA signaling pathway to increase auxin sensitivity. The binding of DELLA and PIF proteins simultaneously results in PIF inactivation. *ATHB2*, *ATHB4*, *REV*, *HFR1*, and *PAR*s are positively regulated by PIFs. HFR1 and PARs bind to PIFs to form non-functional complexes to inhibit the SAS by means of a negative feedback loop. PIFs inhibit the expression of *miR156* to mediate shade-avoidance response (SAS). UVR8 and central clock components PRR proteins negatively regulate SAS through triggering PIF degradation and repressing transcriptional activity of PIF proteins, respectively. Under a shade condition, stable and accumulated TCP17 protein positively regulate SAS *via* activating *PIF4* and *PIF5*. Low blue light levels depressed *CRY1* activity and also increase the abundance of *PIF4* and *PIF5* to trigger hypocotyl elongation with no alteration in detectable auxin amounts or sensitivity. Arrows indicate positive regulation; blunt arrows indicate negative regulation. Pfr, Far-Red light absorbing, biologically active form of phytochrome. Pfr to Pr conversion is optimized by far-red light wavelengths (725–735 nm). Pr, Red-light absorbing, biologically inactive form of phytochrome. Pr to Pfr conversion is optimized by red wavelengths (660–670 nm).

Shade-avoidance responses involve a cascade reaction of the light signal system, plant hormone signaling pathways, and growth regulation (Smith and Whitelam, 1997; Yang and Li, 2017). Current progress in this field of study has mainly focused on the model plant *Arabidopsis* and limited research has been conducted on other plants, especially crops. According to current knowledge, the Pfr of PHYB interacts with PIFs, which are phosphorylated and degraded. As the main switch for the cascade reaction of multiple downstream signals (**Figure 2**), PIFs function by regulating the expression of downstream transcription factors that positively or negatively modulate diverse growth processes, such as earlier flowering, elongation reaction, and branching (Khanna et al., 2004; Leivar et al., 2008; Lorrain et al., 2008; Leivar and Quail, 2011; Li L. et al., 2012).

### PIF Transcription Factors

As transcription factors of the basic helix–loop–helix (b-HLH) family, the prominent function of PIFs is to mediate light signal transduction by interacting with photoreceptors to regulate plant growth and development, such as photomorphogenesis and SAS (Huq and Quail, 2002; Kim et al., 2003; Salter et al., 2003; Huq et al., 2004; Oh et al., 2004; Penfield et al., 2005; Shin et al., 2007). Some PIF proteins, such as PIF1 and PIF3, interact with both PHYB and PHYA, whereas other PIFs, such as PIF4, PIF5, and PIF7, interact preferentially with PHYB (Khanna et al., 2004; Leivar et al., 2008). After interaction with PHYB, PIF3, PIF4, and PIF5 are quickly phosphorylated, then degraded by proteasomes through ubiquitination (Khanna et al., 2004; Leivar et al., 2008; Lorrain et al., 2008). However, unlike its homologs, PIF7 is not rapidly degraded in light (Leivar et al., 2008). Shade treatment rapidly decreases the amount of phosphorylated PIF7 but increases the amount of dephosphorylated PIF7, which is important in the function of PIF7 in regulating the SAS (Li L. et al., 2012). And studies have shown that 14-3-3 proteins negative regulators of the shade response can delay the de-phosphorylation and nuclear import of PIF7 in response to shading (Huang et al., 2018).

PIF4 and PIF5 are positive regulators of the SAS (Lorrain et al., 2008). Compared with the wild type, hypocotyls of the single mutants *pif4* and *pif5* and the double mutant *pif4pif5* display decelerated elongation growth in the case of simulated vegetative shade (Lorrain et al., 2008). Over-expression of full-length or truncated *PIF5* causes the constitutive SAS, even in the absence of shade signal (Lorrain et al., 2008). Perception of low R:FR by the phytochromes stimulates the accumulation of PIF4 and PIF5, which ultimately modulate elongation growth (Lorrain et al., 2008). Similar to PIF4 and PIF5 (Lorrain et al., 2008; Soy et al., 2012), PIF1 and PIF3 are also conducive to SAS, except that the magnitude of their contribution is weaker than that of PIF4 and PIF5 (Leivar et al., 2012a). Within 10 h after treatment with low R:FR illumination, *pif4pif5* double mutants retain shade-avoidance responses to reduced R:FR (Cole et al., 2011), although this shade responsiveness was attenuated in *pif7* mutants (Li L. et al., 2012). Compared with PIF quartet (PIFq; PIF1, PIF3, PIF4, and PIF5) members, PIF7 plays a dominant role in the PHYB-mediated SAS because of the severer shade-defective phenotype of pif7 mutants (Li L. et al., 2012; Mizuno et al., 2015). In addition, PIF7 directly affects the biosynthesis of auxin by activating downstream genes including *YUCCA8* and *YUCCA9* in low R:FR (Li L. et al., 2012). However, the residual responsiveness of *pif7* and *pifq* mutants to low R:FR indicates that other currently unknown pathways or factors control this process. It is worth stating that seedlings of *pif4* and *pif5* fail to elongate under LBL, indicating that PIF4 and PIF5 predominantly mediate responses to LBL (Pedmale et al., 2016).

### Other Regulatory Factors

Overexpression of *PIF3-LIKE 1* (*PIL1*) causes a shift in the biological clock and extreme elongation of hypocotyls (Salter et al., 2003), and these processes are dependent on PIF4 and PIF5 (Salter et al., 2003; Lorrain et al., 2008). During 2 h treatment with low R:FR, the hypocotyl elongation of *pil1* mutants was inhibited (Lorrain et al., 2008), but after continuous treatment for 5 d under low R:FR, significant elongation of hypocotyls was observed (Roig-Villanova et al., 2006). These results indicate that in the regulation of hypocotyl elongation induced by low R:FR, *PIL1* plays both positive and negative roles.

An additional b-HLH gene, *LONG HYPOCOTYL IN FAR-RED LIGHT* (*HFR1*), is quickly up-regulated by low R:FR, and its high expression level is maintained during the following several days (Sessa et al., 2005). Accumulation of HFR1 is induced by prolonged illumination of low R:FR and, subsequently, non-active heterodimers with PIF4 and PIF5 are formed (Sessa et al., 2005; Hornitschek et al., 2009). Compared with the wild type, hypocotyl elongation of *hfr1* mutants is more significantly promoted by low R:FR, whereas transgenic seedlings overexpressing *HFR1* display suppressed hypocotyl elongation (Sessa et al., 2005; Galstyan et al., 2011). Similar to *HFR1*, even with treatment using the protein synthesis inhibitor cycloheximide (CHX), the transcripts of atypical b-HLH transcription factors *PHYTOCHROME RAPIDLY REGULATED 1* (*PAR1*) and *PAR2* are induced quickly and reversibly by low R:FR (Roig-Villanova et al., 2006). These findings indicate that under low R:FR, PIFs activate *HFR1*, *PAR1*, and *PAR2* by means of a negative feedback loop to inhibit the SAS (Zhou et al., 2014). Therefore, *PAR1*, *PAR2*, and *HFR1* are activated by low R:FR, but function negatively by forming a negative feedback loop.

In addition to b-HLH transcription factors (PIFs, *PIL1*, *HFR1*, *PAR1*, and *PAR2*), members of the homeodomain-leucine zipper (HD-Zip) II and HD-Zip III classes of transcription factors are involved in regulation of the SAS (Brandt et al., 2012; Turchi et al., 2015; Merelo et al., 2017). As the first HD-Zip II gene observed to be rapidly and reversibly regulated by changes in R:FR light, *ARABIDOPSIS THALIANA HOMEOBOX 2* (*ATHB2*/*HAT4*) is involved in the elongation response induced by changes in light quality (Carabelli et al., 1993; Carabelli et al., 1996; Steindler et al., 1999). Seedlings with raised levels of *ATHB2* show the elongation response under high R:FR, whereas loss-of-function of *ATHB2* results in an attenuated elongation response under low R:FR (Carabelli et al., 1993; Carabelli et al., 1996; Steindler et al., 1999). PHYB, PHYD, and PHYE are involved in the regulation of *ATHB2* by low R:FR light (Franklin et al., 2003) and *ATHB2* is recognized by PIF5 *in vivo* (Hornitschek et al., 2012). Under a shade environment, inactivation of PHYB increases the stability of PIF proteins, which induces transcriptional expression of *ATHB2* and *ATHB4* (Hornitschek et al., 2012: Gallemí et al., 2017). Subsequently, the elongation reaction of plants is further promoted. Four additional HD-Zip II genes, *HOMEOBOX* ARABIDOPSIS THALIANA *1* (*HAT1*), *HAT2*, *HAT3*, and *ATHB4*, are indicated to be up-regulated by low R:FR (Ciarbelli et al., 2008; Sorin et al., 2009). In addition, under white light, *athb4hat3* mutants do not display a significant difference from the wild type, whereas under low R:FR hypocotyl growth is significantly inhibited compared with that of the wild type (Sorin et al., 2009).

The HD-Zip III transcription factors represented by REVOLUTA have also been shown to positively regulate not only the auxin biosynthesis gene *TRYPTOPHAN AMINOTRANSFERASE OF ARABIDOPSIS 1* (*TAA1*) and *YUCCA*5 (*YUC5*) but also the HD-ZIP II genes *HAT2*, *HAT3*, *ATHB2*/*HAT4*, and *ATHB4* (Brandt et al., 2012). Compared with wild-type plants, *rev* mutants display significantly shorter hypocotyls under low R:FR, whereas seedlings that express functional *REV* exhibit slightly longer hypocotyls in simulated sunlight (Brandt et al., 2012; Merelo et al., 2017). Both HD-Zip II and HD-Zip III proteins are positive regulators of the SAS, but whether they function together in the regulation of downstream gene expression is unknown.

In plants, the TEOSINTE BRANCHED 1, CYCLOIDEA, and PCF (TCP) family of transcription factors perform important functions at diverse stages of plant growth and development, such as leaf morphogenesis, petal development, and flowering (Efroni et al., 2008; Huang and Irish, 2015). It has been recently demonstrated that TCP17 and two additional homologs, TCP5 and TCP13, can activate auxin biosynthesis to initiate hypocotyl elongation induced by shade through both PIF-dependent and -independent pathways. Under constitutive white light, *tcp5 tcp13 tcp17* triple mutants exhibit a slight hypocotyl defective phenotype, whereas under shade hypocotyl elongation of the triple mutants was reduced significantly, suggesting a positive function of TCPs in mediating the SAS (Zhou et al., 2018). Recently, accompanying research findings prove that central clock components PSEUDO-RESPONSE REGULATORS (PRR1/TOC1, PRR5, PRR7, PRR9) negatively regulate shade-avoidance response by directly repressing transcriptional activity of PIF proteins (Franklin, 2020; Zhang et al., 2020). Two other clock rhythm related proteins EARLY FLOWERING 3 (ELF3) and CONSTANS were suggested to regulate shade-avoidance and PIF7 was involved (Jiang et al., 2019; Zhang et al., 2019).

In addition to the afore-mentioned transcription factors, additional proteins and microRNAs are involved in regulation of the SAS. Ectopic expression of the upland cotton gene *FLOWERING PROMOTER FACTOR 1* (*FPF1*) in transgenic *Arabidopsis* results in SAS responses, such as earlier flowering and elongation of petioles and hypocotyls (Wang et al., 2014; Wang et al., 2015). Under low R:FR, accumulated PIF proteins can bind directly to promoters of multiple members of the *MIR156* gene family to inactivate the expression of these *MIR156* genes, thus inducing the expression of *SQUAMOSA PROMOTER-BINDING PROTEIN-LIKE* (*SPL*) genes as the targets of *MIR156* genes. The activated *SPL* genes regulate a suite of essential agronomic traits, such as plant height, branch number, petiole length, leaf number, leaf area, and flowering time (Xie et al., 2017). Recruited by PIF7, *ARABIDOPSIS MORF RELATED GENE 1* (*MRG1*) and *MRG2* were combined with H3K4me3/H3K36me3 to induce histone acetylation and, in this manner, the two genes promote the expression of shade-responsive genes, which include *YUCCA8* and *IAA19* that participate in the biosynthesis and signaling pathways of auxin, and *PACLOBUTRAZOL RESISTANCE1*/*BANQUO1* (*PRE1/BNQ1*), which is involved in brassinosteroid-regulated cell elongation (Peng et al., 2018).

## Roles of Phytohormones in SAS

When plants grow in a shade environment, the perception of PHYB to low R:FR down-regulates the active Pfr form of PHYB. As a result, PIF transcription factors accumulate in the nucleus and control the expression of downstream shade-responsive genes. Low blue light levels depressed CRY1 activity and also increase the abundance of at least PIF4 and PIF5, particularly when combined with low R:FR. In fact, PIFs are emerging as hubs of signal integration, activating several of their targets, including auxin synthesis-related genes, to control plant developmental responses to shade signals (Hornitschek et al., 2012; Li L. et al., 2012; Zhang et al., 2013). Within 1 h of low R:FR treatment, free indole-3-acetic acid (IAA) contents in *Arabidopsis* shoots increased by over 50% (Tao et al., 2008; Li L. et al., 2012; Kohnen et al., 2016). In addition to auxin, gibberellin is also an important hormone involved in SAS (Garcia-Martinez and Gil, 2001; Djakovic-Petrovic et al., 2007; Kurepin et al., 2007a).

### Auxin

Many studies have shown that, as the main natural form of auxin, indole-3-acetic acid (IAA) plays important roles in regulating growth and developmental processes, such as maintenance of apical dominance, responses to light, geotropism, formation of roots and stems, differentiation of vascular bundles, embryonic development, and stem elongation (Zhao, 2018). The gene *SHADE AVOIDANCE 3* (*SAV3*)/*TRYPTOPHAN AMINOTRANSFERASE OF ARABIDOPSIS 1* (*TAA1*) was identified using a forward genetic screen for mutants defective in SAS responses (Tao et al., 2008). Mutation of *SAV3/TAA1* causes insensitivity of hypocotyl elongation to low R:FR illumination (Tao et al., 2008). Predominantly expressed in the cotyledon, *TAA1* encodes a crucial enzyme that catalyzes conversion of tryptophan (Trp) to indole-3-pyruvic acid (IPA) (Stepanova et al., 2008; Zhou et al., 2011). The conversion of IPA to IAA is a rate-limiting step in IAA biosynthesis, which is completed by the catalysis of flavin monooxygenase encoded by *YUCCA* (*YUC*) genes (Won et al., 2011). *YUC2*, *YUC5*, *YUC8*, and *YUC9* are directly activated by PIF4, PIF5, and PIF7, and the *yuc2yuc5yuc8yuc9* deletion mutants were defective in shade-avoidance responses (Hornitschek et al., 2012; Li L. et al., 2012). In hypocotyls, shade induces expression of the auxin output protein PINFORMED 3 (PIN3), and the sensitivity of *pin3-3* mutants to shade is severely impaired (Friml et al., 2002). Additional studies indicate that PIN3-mediated SAS might be universally adapted to shade-intolerant plants, because PIN3 is predominantly localized on the lateral cellular of endodermis cells to form an essential auxin gradient under low R:FR light (Keuskamp et al., 2010).

In addition to the synthesis and transport of auxin, the signaling transduction of auxin also plays an important role in the SAS induced by low R:FR. The deletion mutant *tir1* of the auxin receptor *TRANSPORT INHIBITOR RESPONSE 1* (*TIR1*) is insensitive to low R:FR illumination, and the TIR1 antagonist α-(phenylethyl-2-one)-IAA significantly inhibits hypocotyl elongation under low R:FR (Dharmasiri et al., 2005; Roig-Villanova et al., 2006). In hypocotyls, the high level of PIF4 may induce transcripts of the early auxin-responsive genes *MSG2/IAA19* and *IAA29*, which in turn reduce the expression level of the growth-repressive gene *IAA17* (Hornitschek et al., 2012; Li L. et al., 2012; Pucciariello et al., 2018). Recent studies demonstrate that three auxin-responsive factors (ARF6, ARF7, and ARF8) are conducive to hypocotyl elongation in low R:FR environments (Reed et al., 2018). With regard to PHYB-mediated shade-avoidance responses, auxin contents and auxin-related genes are up-regulated to promote growth (Keuskamp et al., 2010), but hypocotyl elongation triggered by LBL does not involve alteration in detectable auxin amounts or sensitivity (Pedmale et al., 2016). Further evidence suggests that there are two stages in auxin’s roles in SAS (Villanova et al., 2007). During the early hours in shade, the responses are mediated by increased levels of the hormone auxin (Tao et al., 2008; Li L. et al., 2012; Kohnen et al., 2016). But in prolonged shade, the abundance of PIFs, selected auxin receptors and their downstream transcriptional regulators are converted to enhance growth responses, while auxin levels return to those observed before shade (Pucciariello et al., 2018).

### Gibberellin

Gibberellins, first identified in rice, play an important role in regulating diverse developmental processes, such as seed germination, cell elongation, flower induction, and fruit development (Hauvermale et al., 2012). The hypocotyl elongation induced by the *phyB* null mutation in response to low R:FR or LBL is significantly inhibited in a gibberellin-synthesis mutant background (Reed et al., 1996; Djakovic-Petrovic et al., 2007). Furthermore, gibberellin synthesis inhibitor PAC could also inhibit the hypocotyl growth of seedlings in the background of *phyB* mutation, low R:FR or LBL (Reed et al., 1996; Djakovic-Petrovic et al., 2007). Under an identical PAR intensity, in which stem elongation of leguminous plants and oilseed rape is induced in a low R:FR environment, the content of endogenous GAs in the shoot tip is increased (Gawronska et al., 1995; Beall et al., 1996; Potter et al., 1999). GIBBERELLIN 20-OXIDASE 3 (GA20OX3), which is a critical factor involved in GA synthesis, is significantly up-regulated by low R:FR (Devlin et al., 2003). The afore-mentioned results reveal that GA is extremely important for hypocotyl elongation induced by shade-avoidance.

Definitive proof that PHYB affects GA content is presently lacking because GA has various active forms. Compared with the wild type, the contents of some active forms in the *phyB* mutant do not differ significantly, whereas the amounts of certain other active forms are too low to detect (Reed et al., 1996). Although it is unclear whether PHYB regulates GA synthesis, the *phyB* mutant is insensitive to GA treatment (Reed et al., 1996), which indicates that PHYB and GA signals may show a different relationship. After GA binds with the receptor, the activated receptor induces degradation of DELLA growth repressors in the GA signaling pathways. In *Arabidopsis*, the DELLA proteins consist of five members: GIBBERELLIC ACID INSENSITIVE (GAI) (Peng et al., 1997), REPRESSOR OF GA (RGA) (Silverstone et al., 1998), RGA-Like1 (RGL1), RGL2, and RGL3 (Lee et al., 2002; Wen and Chang, 2002; Cheng et al., 2004). The transcript level of *GAI* is up-regulated in a low R:FR environment under the control of PHYB, and is moderated by PHYA (Devlin et al., 2003). Previous studies indicate that *phyB* mutants contain constitutively low contents of RGA and low R:FR results in a sharp (within minutes) decrease in RGA accumulation (Leone et al., 2014). When low R:FR treatment, the petiole length of the quadruple mutant *gai/rga/rgl1/rgl2* does not differ significantly from that of the wild type, but the hypocotyl is longer than that of the wild type (Djakovic-Petrovic et al., 2007). This demonstrates that degradation of DELLA proteins is not necessary for petiole elongation, but the integration of other signaling pathways plays an important role in the regulation of hypocotyl elongation after degradation of DELLA proteins (Djakovic-Petrovic et al., 2007). Accumulation of DELLA proteins abolishes the interaction between PIFs and the promoter of the target gene to suppress PIF transcriptional activity for coordination of hypocotyl elongation (De Lucas et al., 2008; Feng et al., 2008). Additional research suggests that DELLA proteins also stimulate PIF degradation, which is independent of the light-mediated PIF3 degradation pathway, as it can occur in the absence of activated PHYB and the LIGHT-RESPONSE BTB E3 ligase system (Li et al., 2016).

### Brassinosteroid

In *Arabidopsis*, shade-induced hypocotyl elongation was absent in BR biosynthesis mutant *dwarf1* (Luccioni et al., 2002) and *rot3* (Kim et al., 1998), as with wild-type seedlings treated with the BR synthesis inhibitor brassinazole (Keuskamp et al., 2011). Brassinosteroids (BRs) are also essential for petiole growth under low R:FR (Kozuka et al., 2010). Expression of the BR receptor *BRASSINOSTEROID INSENSITIVE 1* (*BRI1*) was up-regulated by low R:FR (Roig-Villanova et al., 2006; Sorin et al., 2009). BRASSINAZOLE-RESISTANT 1 (BZR1), regulating BR signaling pathway, interacts with DELLAs to inhibit the expression of BR-responsive genes (Li Q. F. et al., 2012). BZR1 and PIF4 physically interact and co-regulate their target genes of highly enriched in auxin-responsive and cell wall-related genes, which are repressed by light (Oh et al., 2012; Kohnen et al., 2016). Moreover, the DELLA–BZR1–PIF4 module antagonizes light signaling by activating the auxin signaling and up-regulating the expression of genes related to longitudinal expansion of cells (Casal, 2013; De Lucas and Prat, 2014). Therefore, the module may also play a similar role in responding to shade, but further research is needed. Although certain factors in the BR metabolic pathway are involved in the SAS, the underlying mechanism of their responses to low R:FR or LBL requires further investigation.

### Ethylene

Ethylene, as an endogenous plant-synthesized small molecule, acts at trace levels to regulate diverse developmental processes in plants. Low R:FR increases ethylene concentrations in wild-type tobacco (Pierik et al., 2004). In *Arabidopsis*, shade-induced petiole elongation is absent in the ethylene-insensitive mutants *ein2-1* and *ein3-1eil1-3*, suggesting that ethylene is a positive regulator of shade-induced petiole elongation (Pierik et al., 2009). *In Arabidopsis*, transcription of *1-AMINOCYCLOPROPANE-1-CARBOXYLIC ACID SYNTHASE (ACS2)* is negatively controlled by PHYB (Rodrigues et al., 2014). Compared with wild-type plants, a significantly higher concentration of ethylene is produced in *phyAphyB* mutants, and multiple phenotypes of *phyAphyB* mutants are rescued by application of an ethylene biosynthesis inhibitor (Foo et al., 2006). Similarly, in *Brassica napus BnCRY1*-overexpression seedlings, the transcript levels of *ACS5* and *ACS8* are reduced compared with those of the WT seedlings (Sharma et al., 2014). These results suggest that ethylene synthesis may be negatively regulated by *PHYB* and *CRY1*. Moreover, ethylene promotes hypocotyl elongation by increasing *PIF3* expression in light-grown seedlings (Zhong et al., 2012). In *Arabidopsis*, transcripts of *ACS4* and *ACS8*, which encode critical enzymes in the ethylene biosynthesis pathway, are stimulated by PIF5 (Thain et al., 2004; Khanna et al., 2007). These results suggest an intensive crosstalk between ethylene and PHYB, but the roles of ethylene signal components in SAS are worthy of further studies.

## SAS in Crop

Under high-density planting, the reorientation of leaves towards more light increases individual fitness, but the achievements of elongation growth and accelerated flowering at inappropriate stages are at the expense of leaf area, tiller, and biomass (Donohue et al., 2001; Kebrom and Brutnell, 2007; Carriedo et al., 2016). Although breeders have weakened some of the responses of staple crops by targeting yield, they have not completely eliminated them. A major challenge will be to determine which responses should be manipulated in order to have a significant impact on crop yield, yield stability, crop health, and/or plant quality (Ballaré and Pierik, 2017). Prior to this, the phenotypes and signal transduction mechanisms of different crops in shade need to be clarified. As other plant species, both internodes and petioles of tomato plants are elongated more when exposed to low R:FR. Unlike other species, the size of the shoot apical meristem (SAM), incipient leaf primordia, and the leaf blade of tomato plants are enlarged when exposed to shade. The alteration of leaf morphology has been observed both in cultivated (Stepanova et al., 2011) and wild species (Chitwood et al., 2012). It is shown that low R:FR light produced a typical SAS in *Medicago sativa*, with increased internode and petiole lengths, but unexpectedly with delayed flowering (Christian et al., 2019). Furthermore, a genome-wide expression analysis of rice also uncovered inadequate induction of auxin-responsive genes in the coleoptile when the seedlings were exposed to low R:FR light (Liu et al., 2016). Coincidentally, the Gene Ontology (GO) analysis of maize seedlings exposed to low R:FR light revealed the lack of an enrichment in auxin-responsive genes among those induced by low R:FR light (Wang et al., 2016). Therefore, it is inferred from extensive data collection that auxin response may be a feature of shade-avoidance in dicotyledonous plants, rather than play an important role in monocotyledons (Kurepin et al., 2007b; Procko et al., 2014; Iglesias et al., 2018).

## Discussion

The above findings robustly indicate that multiple photoreceptors as well as several central circadian components connect immediately to downstream transcriptional networks through direct binding to, and repression of, the members of PIF quintet (PIF1, PIF3, PIF4, PIF5, and PIF7) that comprise the signaling hub (Franklin, 2020; Zhang et al., 2020). *Arabidopsis* is an excellent model system to uncover and dissect mechanisms regulating the shade-avoidance responses, some of which are likely to be conserved during evolution. Some important differences are emerging from the analysis of other plant species, so more experimental evidences need to be verified in other plants. Under natural conditions, plants undergo a variety of stress conditions in addition to shade. Low R:FR conditions seem to mostly suppress adaptive responses to phosphate deficiency, drought, pathogens as well as beneficial microbes in the soil (Courbier and Pierik, 2019). Low R:FR can enhance freezing tolerance, but the impact of low R:FR on some environmental stresses may become more aggressive as global temperatures increase (Courbier and Pierik, 2019; Romero-Montepaone et al., 2020). Unraveling the interplay between canopy shade and other stresses, both biotic and abiotic stresses, is beneficial for improving plant fitness and resistance at high planting density. In the future, in addition to further exploration of the regulatory network of shade-avoidance responses, focus on the mechanism of shade-tolerance responses is also required. Although the phenotypic plasticity of shade-tolerant species is low (e.g. scant elongation under low light), the plasticity of some characteristics, especially the morphological characteristics of optimizing light capture, can be high in these plants (Smith, 1982; Valladares and Niinemets, 2008). *Cardamine hirsuta* is a close relative of *Arabidopsis thaliana*, and it is suggested that the lack of a shade-induced hypocotyl elongation response in *C. hirsuta* results from the enhanced repressor activity of the phytochrome A photoreceptor (Molina-Contreras et al., 2019). Exploitation of the molecular basis of shade-avoidance and shade-tolerance to improve crop yield and quality is of considerable importance for high-density cultivation.

## Author Contributions

Conception and design of framework: XW and SF. Data collection: XW, QM, and XG. Analyzed the data: XW and QM. Wrote the paper: XW. Edited the manuscript: QM and YL.

## Funding

The work described in this paper was supported by National Natural Science Foundation Project (31701474) and Henan Science and Technology Project (182102110048).

## Conflict of Interest

The authors declare that the research was conducted in the absence of any commercial or financial relationships that could be construed as a potential conflict of interest.

## References

[B1] Aukerman M. J. Hirschfeld M. Wester L. Weaver M. Clack T. Amasino R. M. (1997). A deletion in the *PHYD* gene of the *Arabidopsis Wassilewskija* ecotype defines a role for phytochrome D in red/far-red light sensing. Plant Cell 9, 1317–1326. 10.1105/tpc.9.8.1317 9286109 PMC157000

[B2] Bae G. Choi G. (2008). Decoding of light signals by plant phytochromes and their interacting proteins. Annu. Rev. Plant Biol. 59, 281–311. 10.1146/annurev.arplant.59.032607.092859 18257712

[B3] Ballaré C. L. Pierik R. (2017). The shade-avoidance syndrome: multiple signals and ecological consequences. Plant Cell Environ. 40, 2530–2543. 10.1111/pce.12914 28102548

[B4] Ballaré C. L. Scopel A. L. Sánchez R. A. (1990). Far-red radiation reflected from adjacent leaves: an early signal of competition in plant canopies. Science 247, 329–332. 10.1126/science.247.4940.329 17735851

[B5] Beall F. D. Yeung E. C. Pharis R. P. (1996). Far-red light stimulates internode elongation, cell division, cell elongation, and gibberellin levels in bean. Can. J. Bot. 74, 743–752. 10.1139/b96-093

[B6] Brandt R. Salla-Martret M. Bou-Torrent J. Musielak T. Stahl M. (2012). Genome-wide binding-site analysis of REVOLUTA reveals a link between leaf patterning and light-mediated growth responses. Plant J. 72, 31–42. 10.1111/j.1365-313X.2012.05049.x 22578006

[B7] Carabelli M. Sessa G. Baima S. Morelli G. Ruberti I. (1993). The *Arabidopsis Athb-2 and-4* genes are strongly induced by far-red-rich light. Plant J. 4, 469–479. 10.1046/j.1365-313X.1993.04030469.x 8106086

[B8] Carabelli M. Morelli G. Whitelam G. Ruberti I. (1996). Twilight-zone and canopy shade induction of the *ATHB-2* homeobox gene in green plants. Proc. Natl. Acad. Sci. U. S. A. 93, 3530–3535. 10.1073/pnas.93.8.3530 11607652 PMC39644

[B9] Carriedo L. G. Maloof J. N. Brady S. M. (2016). Molecular control of crop shade avoidance. Curr. Opin. Plant Biol. 30, 151–158. 10.1016/j.pbi.2016.03.005 27016665

[B10] Casal J. J. (2012). Shade avoidance. Arabidopsis Book 10, e0157. 10.1199/tab.0157 22582029 PMC3350169

[B11] Casal J. J. (2013). Photoreceptor signaling networks in plant responses to shade. Annu. Rev. Plant Biol. 64, 403–427. 10.1146/annurev-arplant-050312-120221 23373700

[B12] Casal J. J. Candia A. N. Sellaro R. (2014). Light perception and signalling by phytochrome A. J. Exp. Bot. 65, 2835–2845. 10.1093/jxb/ert379 24220656

[B13] Cheng H. Qin L. Lee S. Fu X. Richards D. E. Cao D. (2004). Gibberellin regulates *Arabidopsis* floral development via suppression of DELLA protein function. Development 131, 1055–1064. 10.1242/dev.00992 14973286

[B14] Chitwood D. H. Headland L. R. Filiault D. L. Kumar R. Jiménez-Gómez J. M. Schrager A. V. (2012). Native environment modulates leaf size and response to simulated foliar shade across wild tomato species. PloS One 7, e29570. 10.1371/journal.pone.0029570 22253737 PMC3257252

[B15] Chory J. (2010). Light signal transduction: an infinite spectrum of possibilities. Plant J. 61, 982–991. 10.1111/j.1365-313X.2009.04105.x 20409272 PMC3124631

[B16] Christian D. L. Javier A. I. Maximiliano S. L. Mariana S. A. Pedro G. G. (2019). Shade delays flowering in *medicago sativa*. Plant J. 99, 7–22. 10.1111/tpj.14433 30924988

[B17] Ciarbelli A. R. Ciolfi A. Salvucci S. Ruzza V. Possenti M. Carabelli M. (2008). The *Arabidopsis* homeodomain-leucine zipper II gene family: Diversity and redundancy. Plant Mol. Biol. 68, 465–478. 10.1007/s11103-008-9383-8 18758690

[B18] Cole B. Kay S. A. Chory J. (2011). Automated analysis of hypocotyl growth dynamics during shade avoidance in *Arabidopsis*. Plant J. 65, 991–1000. 10.1111/j.1365-313X.2010.04476.x 21288269 PMC3076959

[B19] Courbier S. Pierik R. (2019). Canopy Light Quality Modulates Stress Responses in Plants. iScience 22, 441–452. 10.1016/j.isci.2019.11.035 31816531 PMC6909002

[B20] De Lucas M. Prat S. (2014). PIFs get BRright: PHYTOCHROME INTERACTING FACTORs as integrators of light and hormonal signals. New Phytol. 202, 1126–1141. 10.1111/nph.12725 24571056

[B21] De Lucas M. Daviere J.-M. Rodríguez-Falcón M. Pontin M. Iglesias-Pedraz J. M. Lorrain S. (2008). A molecular framework for light and gibberellin control of cell elongation. Nature 451, 480. 10.1038/nature06520 18216857

[B22] Devlin P. F. Patel S. R. Whitelam G. C. (1998). Phytochrome E influences internode elongation and flowering time in *Arabidopsis*. Plant Cell 10, 1479–1487. 10.2307/3870612 9724694 PMC144080

[B23] Devlin P. F. Yanovsky M. J. Kay S. A. (2003). A genomic analysis of the shade avoidance response in *Arabidopsis*. Plant Physiol. 133, 1617–1629. 10.1104/pp.103.034397 14645734 PMC300718

[B24] Dharmasiri N. Dharmasiri S. Estelle M. (2005). The F-box protein TIR1 is an auxin receptor. Nature 435, 441. 10.1038/nature03543 15917797

[B25] Djakovic-Petrovic T. De Wit M. Voesenek L. A. Pierik R. (2007). DELLA protein function in growth responses to canopy signals. Plant J. 51, 117–126. 10.1111/j.1365-313X.2007.03122.x 17488236

[B26] Donohue K. Pyle E. H. Messiqua D. Heschel M. S. Schmitt J. (2001). Adaptive divergence in plasticity in natural populations of Impatiens capensis and its consequences for performance in novel habitats. Evolution 55, 692–702. 10.2307/2680399 11392387

[B27] Efroni I. Blum E. Goldshmidt A. Eshed Y. (2008). A protracted and dynamic maturation schedule underlies *Arabidopsis* leaf development. Plant Cell 20, 2293–2306. 10.1105/tpc.107.057521 18805992 PMC2570723

[B28] Feng S. Martinez C. Gusmaroli G. Wang Y. Zhou J. Wang F. (2008). Coordinated regulation of *Arabidopsis thaliana* development by light and gibberellins. Nature 451, 475. 10.1038/nature06448 18216856 PMC2562044

[B29] Finlayson S. A. Hays D. B. Morgan P. W. (2007). *phyB-1* sorghum maintains responsiveness to simulated shade, irradiance and red light: far-red light. Plant Cell Environ. 30, 952–962. 10.1111/j.1365-3040.2007.01695.x 17617823

[B30] Foo E. Ross J. J. Davies N. W. Reid J. B. Weller J. L. (2006). A role for ethylene in the phytochrome-mediated control of vegetative development. Plant J. 46, 911–921. 10.1111/j.1365-313X.2006.02754.x 16805726

[B31] Franklin K. A. Quail P. H. (2010). Phytochrome functions in *Arabidopsis* development. J. Exp. Bot. 61, 11–24. 10.1093/jxb/erp304 19815685 PMC2800801

[B32] Franklin K. A. Praekelt U. Stoddart W. M. Billingham O. E. Halliday K. J. Whitelam G. C. (2003). Phytochromes B, D, and E act redundantly to control multiple physiological responses in *Arabidopsis*. Plant Physiol. 131, 1340. 10.1104/pp.102.015487 12644683 PMC166893

[B33] Franklin K. A. (2020). PRR proteins of the circadian clock call time on shade avoidance. Proc. Natl. Acad. Sci. U. S. A, 117, 5095–5096. 10.1073/pnas.2000716117 32060121 PMC7071862

[B34] Friml J. Wiśniewska J. Benková E. Mendgen K. Palme K. (2002). Lateral relocation of auxin efflux regulator PIN3 mediates tropism in *Arabidopsis*. Nature 415, 806. 10.1038/415806a 11845211

[B35] Gallemí M. Contreras M. J. Paulišić S. Salla-Martret M. Sorin C. Godoy M. (2017). A non-DNA-binding activity for the ATHB4 transcription factor in the control of vegetation proximity. New Phytol. 216, 798–813. 10.1111/nph.14727 28805249

[B36] Galstyan A. Cifuentes-Esquivel N. Bou-Torrent J. Martinez-Garcia J. F. (2011). The shade avoidance syndromein *Arabidopsis*: A fundamental role for atypical basic helix-loop-helix proteins as transcriptional cofactors. Plant J. 66, 258–267. 10.1111/j.1365-313X.2011.04485.x 21205034

[B37] Garcia-Martinez J. L. Gil J. (2001). Light regulation of gibberellin biosynthesis and mode of action. J. Plant Growth Regul. 20, 354–368. 10.1007/s003440010033 11986761

[B38] Gawronska H. Yang Y.-Y. Furukawa K. Kendrick R. E. Takahashi N. Kamiya Y. (1995). Effects of low irradiance stress on gibberellin levels in pea seedlings. Plant Cell Physiol. 36, 1361–1367. 10.1093/oxfordjournals.pcp.a078896

[B39] Hauvermale A. L. Ariizumi T. Steber C. M. (2012). Gibberellin signaling: A theme and variations on DELLA repression. Plant Physiol. l160, 83–92. 10.1104/pp.112.200956 PMC3440232 22843665

[B40] Hayes S. Velanis C. N. Jenkins G. I. Franklin K. A. (2014). UV-B detected by the UVR8 photoreceptor antagonizes auxin signaling and plant shade avoidance. Proc. Natl. Acad. Sci. U. S. A. 111, 11894–11899. 10.1073/pnas.1403052111 25071218 PMC4136589

[B41] Hornitschek P. Lorrain S. Zoete V. Michielin O. Fankhauser C. (2009). Inhibition of the shade avoidance response by formation of non-DNA binding bHLH heterodimers. EMBO J. 28, 3893–3902. 10.1038/emboj.2009.306 19851283 PMC2797054

[B42] Hornitschek P. Kohnen M. V. Lorrain S. Rougemont J. Ljung K. López-Vidriero I. (2012). Phytochrome interacting factors 4 and 5 control seedling growth in changing light conditions by directly controlling auxin signaling. Plant J. 71, 699–711. 10.1111/j.1365-313X.2012.05033.x 22536829

[B43] Huang T. Irish V. F. (2015). Temporal control of plant organ growth by TCP transcription factors. Curr. Biol. 25, 1765–1770. 10.1016/j.cub.2015.05.024 26073137

[B44] Huang X. Zhang Q. Jiang Y. Yang C. Wang Q. Li L. (2018). Shade-induced nuclear localization of pif7 is regulated by phosphorylation and 14-3-3 proteins in arabidopsis. eLife 7, e31636. 10.7554/eLife.31636 29926790 PMC6037483

[B45] Huq E. Quail P. H. (2002). *PIF4*, a phytochrome-interacting bHLH factor, functions as a negative regulator of phytochrome B signaling in *Arabidopsis*. EMBO J. 21, 2441–2450. 10.1093/emboj/21.10.2441 12006496 PMC126004

[B46] Huq E. Al-Sady B. Hudson M. Kim C. Apel K. Quail P. H. (2004). Phytochrome-interacting factor 1 is a critical bHLH regulator of chlorophyll biosynthesis. Science 305, 1937–1941. 10.1126/science.1099728 15448264

[B47] Iglesias M. J. Sellaro R. Zurbriggen M. D. Casal J. J. (2018). Multiple links between shade avoidance and auxin networks. J. Exp. Bot. 69, 213–228. 10.1093/jxb/erx295 29036463

[B48] Jarillo J. A. Capel J. Tang R. H. Yang H. Q. Alonso J. M. Ecker J. R. (2001). An Arabidopsis circadian clock component interacts with both CRY1 and phyB. Nature 410, 487–490. 10.1038/35068589 11260718

[B49] Jiang Y. Yang C. Huang S. Xie F. Xu Y. Liu C. (2019). The ELF3-PIF7 Interaction Mediates the Circadian Gating of the Shade Response in Arabidopsis. iScience 22, 288–298. 10.1016/j.isci.2019.11.029 31805433 PMC6909221

[B50] Jumtee K. Okazawa A. Harada K. Fukusaki E. Takano M. Kobayashi A. (2009). Comprehensive metabolite profiling of *phyA phyB phyC* triple mutants to reveal their associated metabolic phenotype in rice leaves. J. Biosci. Bioeng. 108, 151–159. 10.1016/j.jbiosc.2009.03.010 19619864

[B51] Kay S. A. Shinozaki K. Chua N.-H. (1989). The sequence of the rice phytochrome gene. Nucleic Acids Res. 17, 2865–2866. 10.1093/nar/17.7.2865 2717416 PMC317674

[B52] Kebrom T. H. Brutnell T. P. (2007). The molecular analysis of the shade avoidance syndrome in the grasses has begun. J. Exp. Bot. 58, 3079–3089. 10.1093/jxb/erm205 17921475

[B53] Kebrom T. H. Brutnell T. P. Finlayson S. A. (2010). Suppression of sorghum axillary bud outgrowth by shade, *phyB* and defoliation signalling pathways. Plant Cell Environ. 33, 48–58. 10.1111/j.1365-3040.2009.02050.x 19843258

[B54] Keuskamp D. H. Pollmann S. Voesenek L. A. Peeters A. J. Pierik R. (2010). Auxin transport through PIN-FORMED 3 (PIN3) controls shade avoidance and fitness during competition. Proc. Natl. Acad. Sci. U. S. A. 107, 22740–22744. 10.1073/pnas.1013457108 21149713 PMC3012496

[B55] Keuskamp D. H. Sasidharan R. Vos I. Peeters A. J. Voesenek L. A. Pierik R. (2011). Blue-light-mediated shade avoidance requires combined auxin and brassinosteroid action in Arabidopsis seedlings. Plant J. 67, 208–217. 10.1111/j.1365-313X.2011.04597.x 21457374

[B56] Kevei E. Gyula P. Hall A. Kozma-Bognar L. Kim W. Y. Eriksson M. E. (2006). Forward genetic analysis of the circadian clock separates the multiple functions of ZEITLUPE. Plant Physiol. 140, 933–945. 10.1104/pp.105.074864 16428597 PMC1400575

[B57] Khanna R. Huq E. Kikis E. A. Al-Sady B. Lanzatella C. Quail P. H. (2004). A novel molecular recognition motif necessary for targeting photoactivated phytochrome signaling to specific basic helix-loop-helix transcription factors. Plant Cell 16, 3033–3044. 10.1105/tpc.104.025643 15486100 PMC527196

[B58] Khanna R. Shen Y. Marion C. M. Tsuchisaka A. Theologis A. Schäfer E. (2007). The basic helix-loop-helix transcription factor PIF5 acts on ethylene biosynthesis and phytochrome signaling by distinct mechanisms. Plant Cell 19, 3915–3929. 10.1105/tpc.107.051508 18065691 PMC2217642

[B59] Kim G. T. Tsukaya H. Uchimiya H. (1998). The *ROTUNDIFOLIA3* gene of *Arabidopsis thaliana* encodes a new member of the cytochrome P-450 family that is required for the regulated polar elongation of leaf cells. Genes Dev. 12, 2381–2391. 10.1101/gad.12.15.2381 9694802 PMC317051

[B60] Kim J. Yi H. Choi G. Shin B. Song P. S. Choi G. (2003). Functional characterization of phytochrome interacting factor 3 in phytochrome-mediated light signal transduction. Plant Cell 15, 2399–2407. 10.1105/tpc.014498 14508006 PMC197304

[B61] Kohnen M. V. Schmid-Siegert E. Trevisan M. Petrolati L. A. Senechal F. Müller-Moulé P. (2016). Neighbor detection induces organ-specific transcriptomes, revealing patterns underlying hypocotyl-specific growth. Plant Cell 28, 2889–2904. 10.1105/tpc.16.00463 27923878 PMC5240736

[B62] Kozuka T. Kobayashi J. Horiguchi G. Demura T. Sakakibara H. Tsukaya H. (2010). Involvement of auxin and brassinosteroid in the regulation of petiole elongation under the shade. Plant Physiol. 153, 1608–1618. 10.1104/pp.110.156802 20538889 PMC2923899

[B63] Kurepin L. V. Emery R. J. Pharis R. P. Reid D. M. (2007a). The interaction of light quality and irradiance with gibberellins, cytokinins and auxin in regulating growth of *Helianthus annuus* hypocotyls. Plant Cell Environ. 30, 147–155. 10.1111/j.1365-3040.2006.01612.x 17238906

[B64] Kurepin L. V. Emery R. J. Pharis R. P. Reid D. M. (2007b). Uncoupling light quality from light irradiance effects in *Helianthus annuus* shoots: Putative roles for plant hormones in leaf and internode growth. J. Exp. Bot. 58, 2145–2157. 10.1093/jxb/erm068 17490995

[B65] Lee S. Cheng H. King K. E. Wang W. He Y. Hussain A. (2002). Gibberellin regulates *Arabidopsis* seed germination via RGL2, a GAI/RGA-like gene whose expression is up-regulated following imbibition. Genes Dev. 16, 646–658. 10.1101/gad.969002 11877383 PMC155355

[B66] Leivar P. Quail P. H. (2011). PIFs: pivotal components in a cellular signaling hub. Trends Plant Sci. 16, 19–28. 10.1016/j.tplants.2010.08.003 20833098 PMC3019249

[B67] Leivar P. Monte E. Sady B. Carle C. Storer A. Alonso J. M. (2008). The *Arabidopsis* phytochrome-interacting factor PIF7, together with PIF3 and PIF4, regulates responses to prolonged red light by modulating phyB levels. Plant Cell 20, 337–352. 10.1105/tpc.107.052142 PMC2276449 18252845

[B68] Leivar P. Tepperman J. M. Cohn M. M. Monte E. Al-Sady B. Erickson E. (2012). Dynamic antagonism between phytochromes and PIF family basic helix-loop-helix factors induces selective reciprocal responses to light and shade in a rapidly responsive transcriptional network in *Arabidopsis*. Plant Cell 24, 1398–1419. 10.1105/tpc.112.095711 22517317 PMC3398554

[B69] Leone M. Keller M. M. Cerrudo I. Ballare C. L. (2014). To grow or defend? Low red : far-red ratios reduce jasmonate sensitivity in *Arabidopsis* seedlings by promoting DELLA degradation and increasing JAZ10 stability. New Phytol. 204, 355–367. 10.1111/nph.12971 25103816

[B70] Li L. Ljung K. Breton G. Schmitz R. J. Pruneda-Paz J. Zitron C. (2012). Linking photoreceptor excitation to changes in plant architecture. Genes Dev. 26, 785–790. 10.1101/gad.187849.112 22508725 PMC3337452

[B71] Li Q. F. Wang C. Jiang L. Li S. Sun S. S. He J. X. (2012). An interaction between BZR1 and DELLAs mediates direct signaling crosstalk between brassinosteroids and gibberellins in *Arabidopsis*. Sci. Signal. 5, ra72–ra72. 10.1126/scisignal.2002908 23033541

[B72] Li K. Yu R. Fan L. M. Wei N. Chen H. Deng X. W. (2016). DELLA-mediated PIF degradation contributes to coordination of light and gibberellin signalling in *Arabidopsis*. Nat. Commun. 7, 11868. 10.1038/ncomms11868 27282989 PMC4906400

[B73] Liu H. Yang C. Li L. (2016). Shade-induced stem elongation in rice seedlings: Implication of tissue-specific phytohormone regulation. J. Integr. Plant Biol. 58, 614–617. 10.1111/jipb.12468 26888633

[B74] Lorrain S. Allen T. Duek P. D. Whitelam G. C. Fankhauser C. (2008). Phytochrome-mediated inhibition of shade avoidance involves degradation of growth-promoting bHLH transcription factors. Plant J. 53, 312–323. 10.1111/j.1365-313X.2007.03341.x 18047474

[B75] Losi A. Gärtner W. (2012). The evolution of flavin-binding photoreceptors: an ancient chromophore serving trendy blue-light sensors. Annu. Rev. Plant Biol. 63, 49–72. 10.1146/annurev-arplant-042811-105538 22136567

[B76] Luccioni L. G. Oliverio K. A. Yanovsky M. J. Boccalandro H. E. Casal J. J. (2002). Brassinosteroid mutants uncover fine tuning of phytochrome signaling. Plant Physiol. 128, 173–181. 10.1104/pp.010668 11788763 PMC148967

[B77] Martinez-Garcia J. F. Gallemi M. Molina-Contreras M. J. Llorente B. Bevilaqua M. R. Quail P. H. (2014). The shade avoidance syndrome in *Arabidopsis*: the antagonistic role of phytochrome a and b differentiates vegetation proximity and canopy shade. PloS One 9, e109275. 10.1371/journal.pone.0109275 25333270 PMC4204825

[B78] Mathews S. Sharrock R. (1997). Phytochrome gene diversity. Plant Cell Environ. 20, 666–671. 10.1046/j.1365-3040.1997.d01-117.x

[B79] Mazza C. A. Ballaré C. L. (2015). Photoreceptors UVR8 and phytochrome B cooperate to optimize plant growth and defense in patchy canopies. New Phytol. 207, 4–9. 10.1111/nph.13332 25659974

[B80] Merelo P. Paredes E. B. Heisler M. G. Wenkel S. (2017). The shady side of leaf development: The role of the REVOLUTA/KANADI1 module in leaf patterning and auxin-mediated growth promotion. Curr. Opin. Plant Biol. 35, 111–116. 10.1016/j.pbi.2016.11.016 27918939

[B81] Middleton L. (2001). Shade-tolerant flowering plants: adaptations and horticultural implications. Acta Hortic. 552, 95–102. 10.17660/ActaHortic.2001.552.9

[B82] Mizuno T. Oka H. Yoshimura F. Ishida K. Yamashino T. (2015). Insight into the mechanism of end-of-day far-red light (EODFR)-induced shade avoidance responses in *Arabidopsis thaliana*. Biosci. Biotechnol. Biochem. 79, 1987–1994. 10.1080/09168451.2015.1065171 26193333

[B83] Molina-Contreras M. J. Paulišić S. Then C. Moreno-Romero J. Pastor-Andreu P. (2019). Photoreceptor Activity Contributes to Contrasting Responses to Shade in Cardamine and Arabidopsis Seedlings. Plant Cell 31, 2649–2663. 10.1105/tpc.19.00275 31530733 PMC6881134

[B84] Morgan D. Smith H. (1978). The relationship between phytochrome-photoequilibrium and Development in light grown *Chenopodiumalbum* L. Planta 142, 187–193. 10.1007/BF00388211 24408101

[B85] Oh E. Kim J. Park E. Kim J. I. Kang C. Choi G. (2004). PIL5, a phytochrome-interacting basic helix-loop-helix protein, is a key negative regulator of seed germination in *Arabidopsis thaliana*. Plant Cell 16, 3045–3058. 10.1105/tpc.104.025163 15486102 PMC527197

[B86] Oh E. Zhu J.-Y. Wang Z.-Y. (2012). Interaction between BZR1 and PIF4 integrates brassinosteroid and environmental responses. Nat. Cell Biol. 14, 802. 10.1038/ncb2545 22820378 PMC3703456

[B87] Pedmale U. V. Huang S. S. C. Zander M. Cole B. J. Hetzel J. Ljung K. (2016). Cryptochromes interact directly with PIFs to control plant growth in limiting blue light. Cell 164, 233–245. 10.1016/j.cell.2015.12.018 26724867 PMC4721562

[B88] Penfield S. Josse E. M. Kannangara R. Gilday A. D. Halliday K. J. Graham I. A. (2005). Cold and light control seed germination through the bHLH transcription factor spatula. Curr. Biol. 15, 1998–2006. 10.1016/j.cub.2005.11.010 16303558

[B89] Peng J. Carol P. Richards D. E. King K. E. Cowling R. J. Murphy G. P. (1997). The *Arabidopsis* GAI gene defines a signaling pathway that negatively regulates gibberellin responses. Genes Dev. 11, 3194–3205. 10.1101/gad.11.23.3194 9389651 PMC316750

[B90] Peng M. Li Z. Zhou N. Ma M. Jiang Y. (2018). Linking phytochrome-interacting factor to histone modification in plant shade avoidance. Plant Physiol. 176, 1341–1351. 10.1104/pp.17.01189 29187567 PMC5813548

[B91] Pierik R. Cuppens M. L. Voesenek L. A. Visser E. J. (2004). Interactions between ethylene and gibberellins in phytochrome-mediated shade avoidance responses in tobacco. Plant Physiol. 136, 2928–2936. 10.1104/pp.104.045120 15448197 PMC523355

[B92] Pierik R. Djakovic-Petrovic T. Keuskamp D. H. de Wit M. Voesenek L. A. (2009). Auxin and ethylene regulate elongation responses to neighbor proximity signals independent of gibberellin and della proteins in Arabidopsis. Plant Physiol. 149, 1701–1712. 10.1104/pp.108.133496 19211699 PMC2663759

[B93] Potter T. I. Rood S. B. Zanewich K. P. (1999). Light intensity, gibberellin content and the resolution of shoot growth in *Brassica*. Planta 207, 505–511. 10.2307/23385597

[B94] Procko C. Crenshaw C. M. Ljung K. Noel J. P. Chory J. (2014). Cotyledon-generated auxin is required for shade-induced hypocotyl growth in *Brassica rapa*. Plant Physiol. 165, 1285–1301. 10.1104/pp.114.241844 24891610 PMC4081337

[B95] Pucciariello O. Legris M. Costigliolo Rojas C. Iglesias M. J. Hernando C. E. Dezar C. (2018). Rewiring of auxin signaling under persistent shade. Proc. Natl. Acad. Sci. U.S.A. 115, 5612–5617. 10.1073/pnas.1721110115 29724856 PMC6003476

[B96] Rausenberger J. Tscheuschler A. Nordmeier W. Wust F. Timmer J. Schäfer E. (2011). Photoconversion and nuclear trafficking cycles determine phytochrome a\’s response profile to far-red light. Cell 146, 813–825. 10.1016/j.cell.2011.07.023 21884939

[B97] Reed J. W. Nagpal P. Poole D. S. Furuya M. Chory J. (1993). Mutations in the gene for the red/far-red light receptor phytochrome B alter cell elongation and physiological responses throughout Arabidopsis development. Plant Cell 5, 147–157. 10.1105/tpc.5.2.147 8453299 PMC160258

[B98] Reed J. W. Foster K. R. Morgan P. W. Chory J. (1996). Phytochrome b affects responsiveness to gibberellins in *Arabidopsis*. Plant Physiol. 112, 337–342. 10.1104/pp.112.1.337 8819329 PMC157954

[B99] Reed J. W. Wu M. F. Reeves P. H. Hodgens C. Yadav V. Hayes S. (2018). Three Auxin Response Factors Promote Hypocotyl Elongation. Plant Physiol. 2, 864–875. 10.1104/pp.18.00718 PMC6181040 30139794

[B100] Rizzini L. Favory J.-J. Cloix C. Faggionato D. O’hara A. Kaiserli E. (2011). Perception of UV-B by the *Arabidopsis* UVR8 protein. Science 332, 103–106. 10.1126/science.1200660 21454788

[B101] Rodrigues M. A. Bianchetti R. E. Freschi L. (2014). Shedding light on ethylene metabolism in higher plants. Front. Plant Sci. 5, 665. 10.3389/fpls.2014.00665 25520728 PMC4249713

[B102] Roig-Villanova I. Bou J. Sorin C. Devlin P. F. Martínez-García J. F. (2006). Identification of primary target genes of phytochrome signaling. Early transcriptional control during shade avoidance responses in *Arabidopsis*. Plant Physiol. 141, 85–96. 10.1104/pp.105.076331 16565297 PMC1459307

[B103] Romero-Montepaone S. Poodts S. Fischbach P. Sellaro R. Zurbriggen M. D. Casal J. J. (2020). Shade-avoidance responses become more aggressive in warm environments. Plant Cell Environ. in press. 10.1111/pce.13720 31925796

[B104] Salter M. G. Franklin K. A. Whitelam G. C. (2003). Gating of the rapid shade-avoidance response by the circadian clock in plants. Nature 426, 680. 10.1038/nature02174 14668869

[B105] Sellaro R. Crepy M. Trupkin S. A. Karayekov E. Buchovsky A. S. Rossi C. (2010). Cryptochrome as a sensor of the blue/green ratio of natural radiation in *Arabidopsis*. Plant Physiol. 154, 401–409. 10.1104/pp.110.160820 20668058 PMC2938137

[B106] Sellaro R. Yanovsky M. J. Casal J. J. (2011). Repression of shade-avoidance reactions by sunfleck induction of HY5 expression in *Arabidopsis*. Plant J. Cell Mol. Biol. 68, 919–928. 10.1111/j.1365-313X.2011.04745.x 21848684

[B107] Sessa G. Carabelli M. Sassi M. Ciolfi A. Possenti M. Mittempergher F. (2005). A dynamic balance between gene activation and repression regulates the shade avoidance response in *Arabidopsis*. Gene Dev. 19, 2811–2815. 10.1101/gad.364005 16322556 PMC1315388

[B108] Sharma P. Chatterjee M. Burman N. Khurana J. P. (2014). Cryptochrome 1 regulates growth and development in Brassica through alteration in the expression of genes involved in light, phytohormone and stress signalling. Plant Cell Environ. 37, 961–977. 10.1111/pce.12212 24117455

[B109] Sharma A. Sharma B. Hayes S. Kerner K. Hoecker U. Jenkins G. I. (2019). UVR8 disrupts stabilisation of PIF5 by COP1 to inhibit plant stem elongation in sunlight. Nat. Commun. 10, 1–10. 10.1038/s41467-019-12369-1 31562307 PMC6764944

[B110] Sheehan M. J. Farmer P. R. Brutnell T. P. (2004). Structure and expression of maize phytochrome family homeologs. Genetics 167, 1395–1405. 10.1534/genetics.103.026096 15280251 PMC1470959

[B111] Sheehan M. J. Kennedy L. M. Costich D. E. Brutnell T. P. (2007). Subfunctionalization of PhyB1 and PhyB2 in the control of seedling and mature plant traits in maize. Plant J. 49, 338–353. 10.1111/j.1365-313X.2006.02962.x 17181778

[B112] Shin J. Park E. Choi G. (2007). PIF3 regulates anthocyanin biosynthesis in an HY5-dependent manner with both factors directly binding anthocyanin biosynthetic gene promoters in *Arabidopsis*. Plant J. 49, 981–994. 10.1111/j.1365-313X.2006.03021.x 17319847

[B113] Shinomura T. Uchida K. Furuya M. (2000). Elementary processes of photoperception by phytochrome a for high-irradiance response of hypocotyl elongation in *Arabidopsis*. Plant Physiol. 122, 147–156. 10.1104/pp.122.1.147 10631258 PMC58853

[B114] Silverstone A. L. Ciampaglio C. N. Sun T. (1998). The *Arabidopsis* RGA gene encodes a transcriptional regulator repressing the gibberellin signal transduction pathway. Plant Cell 10, 155–169. 10.1105/tpc.10.2.155 9490740 PMC143987

[B115] Smith H. Whitelam G. (1997). The shade avoidance syndrome: multiple responses mediated by multiple phytochromes. Plant Cell Environ. 20, 840–844. 10.1046/j.1365-3040.1997.d01-104.x

[B116] Smith H. (1982). Light quality, photoperception, and plant strategy. Annu. Rev. Plant Physiol. 33, 481–518. 10.1146/annurev.pp.33.060182.002405

[B117] Somers D. E. Schultz T. F. Milnamow M. Kay S. A. (2000). ZEITLUPE encodes a novel clock-associated PAS protein from Arabidopsis. Cell 101, 319–329. 10.1016/S0092-8674(00)80841-7 10847686

[B118] Sorin C. L. Salla-Martret M. Bou-Torrent J. Roig-Villanova I. Martı´Nez-Garc´ıa J. F. (2009). ATHB4, a regulator of shade avoidance, modulates hormone response in *Arabidopsis* seedlings. Plant J. 10.1111/j.1365-313X.2009.03866.x 19392702

[B119] Soy J. Leivar P. Gonzalez-Schain N. Sentandreu M. Prat S. Quail P. H. (2012). Phytochrome-imposed oscillations in PIF3 protein abundance regulate hypocotyl growth under diurnal light/dark conditions in *Arabidopsis*. Plant J. 71, 390–401. 10.1111/j.1365-313X.2012.04992.x 22409654 PMC3465574

[B120] Steindler C. Matteucci A. Sessa G. Weimar T. Ohgishi M. Aoyama T. (1999). Shade avoidance responses are mediated by the ATHB-2 HD-Zip protein, a negative regulator of gene expression. Development 125, 4235–4245. 10.1007/s004290050293 10477292

[B121] Stepanova A. N. Robertson-Hoyt J. Yun J. Benavente L. M. Xie D.-Y. Doležal K. (2008). TAA1-mediated auxin biosynthesis is essential for hormone crosstalk and plant development. Cell 133, 177–191. 10.1016/j.cell.2008.01.047 18394997

[B122] Stepanova A. N. Yun J. Robles L. M. Novak O. He W. Guo H. (2011). The Arabidopsis YUCCA1 flavin monooxygenase functions in the indole-3-pyruvic acid branch of auxin biosynthesis. Plant Cell 23, 3961–3973. 10.1105/tpc.111.088047 22108406 PMC3246335

[B123] Sun W. Xu X. H. Wu X. Wang Y. Lu X. Sun H. (2015). Genome-wide identification of microRNAs and their targets in wild type and phyB mutant provides a key link between microRNAs and the phyB-mediated light signaling pathway in rice. Front. Plant Sci. 6, 372. 10.3389/fpls.2015.00372 26074936 PMC4448008

[B124] Tao Y. Ferrer J.-L. Ljung K. Pojer F. Hong F. Long J. A. (2008). Rapid synthesis of auxin via a new tryptophan-dependent pathway is required for shade avoidance in plants. Cell 133, 164–176. 10.1016/j.cell.2008.01.049 18394996 PMC2442466

[B125] Thain S. C. Vandenbussche F. Laarhoven L. J. Dowson-Day M. J. Wang Z. Y. Tobin E. M. (2004). Circadian rhythms of ethylene emission in. Arabidopsis Plant Physiol. 136, 3751–3761. 10.1104/pp.104.042523 15516515 PMC527172

[B126] Turchi L. Baima S. Morelli G. Ruberti I. (2015). Interplay of HD-Zip II and III transcription factors inauxin-regulated plant development. J. Exp. Bot. 66, 5043–5053. 10.1093/jxb/erv174 25911742

[B127] Valladares F. Niinemets U. (2008). Shade Tolerance, a Key Plant Feature of Complex Nature and Consequences. Annu. Rev. Ecol. Evol. Syst. 39, 237–257. 10.1146/annurev.ecolsys.39.110707.173506

[B128] Villanova I. Torrent J. Galstyan A. Paulet L. Portolés S. Concepción M. (2007). Interaction of shade avoidance and auxin responses: a role for two novel atypical bHLH proteins. The EMBO Journal 26, 4756–4767. 10.1038/sj.emboj.7601890 17948056 PMC2080812

[B129] Wang X. Fan S. Song M. Pang C. Wei H. Yu J. (2014). Upland cotton gene *GhFPF1* confers promotion of flowering time and shade-avoidance responses in *Arabidopsis thaliana*. PloS One 9, e91869. 10.1371/journal.pone.0091869 24626476 PMC3953518

[B130] Wang X. Pang C. Wei H. Yu S. (2015). Involvement of cotton gene *GhFPF1* in the regulation of shade avoidance responses in *Arabidopsis thaliana*. Plant Signal. Behav. 10, e1062195. 10.1080/15592324.2015.1062195 26337193 PMC4883930

[B131] Wang H. Wu G. Zhao B. Wang B. Lang Z. Zhang C. (2016). Regulatory modules controlling early shade avoidance response in maize seedlings. BMC Genom. 17, 269. 10.1186/s12864-016-2593-6 PMC4815114 27030359

[B132] Wen C. K. Chang C. (2002). *Arabidopsis* RGL1 encodes a negative regulator of gibberellin responses. Plant Cell 14, 87–100. 10.1105/tpc.010325 11826301 PMC150553

[B133] Won C. Shen X. Mashiguchi K. Zheng Z. Dai X. Cheng Y. (2011). Conversion of tryptophan to indole-3-acetic acid by tryptophan aminotransferases of *Arabidopsis* and YUCCAs in *Arabidopsis*. Proc. Natl. Acad. Sci. 108, 18518–18523. 10.1073/pnas.1108436108 22025721 PMC3215067

[B134] Xie Y. Liu Y. Wang H. Ma X. Wang B. Wu G. (2017). Phytochrome-interacting factors directly suppress MIR156 expression to enhance shade-avoidance syndrome in *Arabidopsis*. Nat. Commun. 8, 348. 10.1038/s41467-017-00404-y 28839125 PMC5570905

[B135] Yang C. Li L. (2017). Hormonal Regulation in Shade Avoidance. Front. Plant Sci. 8, 1527. 10.3389/fpls.2017.01527 28928761 PMC5591575

[B136] Yanovsky M. J. Casal J. J. Whitelam G. C. (1995). Phytochrome A, phytochrome B and HY4 are involved in hypocotyl growth responses to natural radiation in *Arabidopsis*: weak de-etiolation of the *phyA* mutant under dense canopies. Plant Cell Environ. 18, 788–794. 10.1111/j.1365-3040.1995.tb00582.x

[B137] Yu X. Klejnot J. Zhao X. Shalitin D. Maymon M. Yang H. (2007). *Arabidopsis* cryptochrome 2 completes its posttranslational life cycle in the nucleus. Plant Cell 19, 3146–3156. 10.1105/tpc.107.053017 17965271 PMC2174722

[B138] Zhang Y. Mayba O. Pfeiffer A. Shi H. Tepperman J. M. (2013). A quartet of PIF bHLH factors provides a transcriptionally centered signaling hub that regulates seedling morphogenesis through differential expression-patterning of shared target genes in Arabidopsis. PloS Genet. 9, e1003244. 10.1371/journal.pgen.1003244 23382695 PMC3561105

[B139] Zhang R. Yang C. Jiang Y. Li L. (2019). A PIF7-CONSTANS-Centered Molecular Regulatory Network Underlying Shade-Accelerated Flowering. Mol. Plant 12, 1587–1597. 10.1016/j.molp.2019.09.007 31568831

[B140] Zhang Y. Pfeiffer A. Tepperman J. M. Dalton-Roesler J. Leivar P. Grandio E. G. (2020). Central clock components modulate plant shade avoidance by directly repressing transcriptional activation activity of PIF proteins. Proc. Natl. Acad. Sci. U. S. A. 6, 3261–3269. 10.1073/pnas.1918317117 PMC7022205 31988133

[B141] Zhao Y. (2018). Essential roles of local auxin biosynthesis in plant development and in adaptation to environmental changes. Annu. Rev. Plant Biol. 69, 417–435. 10.1146/annurev-arplant-042817-040226 29489397

[B142] Zhong S. Shi H. Xue C. Wang L. Xi Y. Li J. (2012). A molecular framework of light-controlled phytohormone action in *Arabidopsis*. Curr. Biol. 22, 1530–1535. 10.1016/j.cub.2012.06.039 22818915 PMC4437768

[B143] Zhou Z. Y. Zhang C. G. Wu L. Zhang C. G. Chai J. Wang M. (2011). Functional characterization of the CKRC1/TAA1 gene and dissection of hormonal actions in the *Arabidopsis* root. Plant J. 66, 516–527. 10.1111/j.1365-313X.2011.04509.x 21255165

[B144] Zhou P. Song M. Yang Q. Su L. Hou P. Guo L. (2014). Both PHYTOCHROME RAPIDLY REGULATED1 (PAR1) and PAR2 promote seedling photomorphogenesis in multiple light signaling pathways. Plant Physiol. 164, 841–852. 10.1104/pp.113.227231 24335334 PMC3912110

[B145] Zhou Y. Zhang D. An J. Yin H. Fang S. Chu J. (2018). TCP transcription factors regulate shade avoidance via directly mediating the expression of both phytochrome interacting factors and auxin biosynthetic genes. Plant Physiol. 176, 1850–1861. 10.1104/pp.17.01566 29254986 PMC5813557

[B146] Zou Y. Li R. Baldwin I. T. (2019). ZEITLUPE is required for shade avoidance in the wild tobacco *Nicotiana attenuata*. J. Integr. Plant Biol. 0, 1–11. 10.1111/jipb.12880 31628717

